# The Degradation of Hyaluronan in the Skin

**DOI:** 10.3390/biom12020251

**Published:** 2022-02-03

**Authors:** Petra Žádníková, Romana Šínová, Vojtěch Pavlík, Matěj Šimek, Barbora Šafránková, Martina Hermannová, Kristina Nešporová, Vladimír Velebný

**Affiliations:** 1 R&D Department, Contipro a.s., 561 02 Dolní Dobrouč, Czech Republic; zadnikova@contipro.com (P.Ž.); romana.sinova@contipro.com (R.Š.); vojtech.pavlik@contipro.com (V.P.); matej.simek@contipro.com (M.Š.); barbora.safrankova@contipro.com (B.Š.); martina.hermannova@contipro.com (M.H.); vladimir.velebny@contipro.com (V.V.); 2 Faculty of Medicine in Hradec Králové, Charles University, 500 03 Hradec Králové, Czech Republic; 3 Institute of Experimental Biology, Faculty of Science, Masaryk University, 611 37 Brno, Czech Republic; 4 Third Faculty of Medicine, Charles University, 100 00 Prague, Czech Republic

**Keywords:** hyaluronan, degradation, skin, hyaluronidase, CEMIP, TMEM2

## Abstract

Hyaluronan (HA) comprises a fundamental component of the extracellular matrix and participates in a variety of biological processes. Half of the total amount of HA in the human body is present in the skin. HA exhibits a dynamic turnover; its half-life in the skin is less than one day. Nevertheless, the specific participants in the catabolism of HA in the skin have not yet been described in detail, despite the essential role of HA in cutaneous biology. A deeper knowledge of the processes involved will act to support the development of HA-based topical and implantable materials and enhance the understanding of the various related pathological cutaneous conditions. This study aimed to characterize the distribution and activity of hyaluronidases and the other proteins involved in the degradation of HA in healthy human full-thickness skin, the epidermis and the dermis. Hyaluronidase activity was detected for the first time in healthy human skin. The degradation of HA occurred in lysates at an acidic pH. HA gel zymography revealed a single band corresponding to approximately 50 kDa. This study provided the first comprehensive view of the distribution of canonic HA-degrading proteins (HYAL1 and HYAL2) in human skin employing IHF and IHC. Furthermore, contrary to previous assumptions TMEM2, a novel hyaluronidase, as well as CEMIP, a protein involved in HA degradation, were localized in the human epidermis, as well as in the dermis.

## 1. Introduction

Hyaluronan or hyaluronic acid (HA) comprises a negatively charged glycosaminoglycan (GAG) that is made up of repeating disaccharide units (-d-glucuronic β1,3-*N*-acetyl-d-glucosamine [β1, 4-]) that may attain as high as 10^7^ Daltons. It is the only non-sulfated GAG, and it lacks a covalent attachment to proteoglycan core proteins [1,2]. HA is distributed ubiquitously throughout the extracellular matrix (ECM) of the skin [3]. HA retains water to a remarkable extent; it is responsible for the hydration and viscoelasticity of the skin and facilitates the transport of ion solutes and nutrients. In view of these roles, HA was initially considered to comprise merely an inert component of the ECM. However, it has been demonstrated that HA actively participates in cell motility, embryonic development, malignant progression, tissue repair and regeneration, angiogenesis and immune response [4,5,6]. Hyaluronan protects the skin from free radical damage, particularly via the sequestration of ROS generated by UV radiation [7]. 

The metabolism of HA is highly dynamic [8]. The half-life of epidermal HA is 2–3 h and degradation occurs in situ [9], while HA has a half-life of approximately 1 day in the dermis and is degraded both in situ and systemically [10,11]; the latter elimination path also requires an initial fragmentation of the HA prior to drainage into the vessels [12,13]. 

The human genome encodes six hyaluronidase-like genes. HYAL1 is an acid-active (pH optimum 3.5) lysosomal enzyme. It binds and degrades HA of any size, and it generates hexasaccharides and, predominantly, tetrasaccharides (0.8 kDa) [14]. HYAL2 is acid active (a pH optimum of below 4, [15]) and has two forms, one of which is anchored to the extracellular surface of the plasma membrane. This HYAL2 form acts to degrade high-molecular-weight hyaluronan (HMW HA) into intermediate-sized fragments [16]. The other HYAL2 form is soluble and cleaves HA in lysosomes to 20 kDa oligomers [17]. Testicular HYAL3 has also been detected in other somatic tissues; however, it lacks any detectable activity in vitro, while HYAL4 cleaves chondroitin sulfate and does not cleave HA. PH20 is expressed in sperm; it cleaves HMW HA and is active at a neutral pH [18]. Therefore, PH20, HYAL3 and HYAL4 are unlikely to influence the degradation of HA in the skin [19], while HYAL1 and HYAL2 most likely play key roles in the catabolism of HA in somatic tissues [20]. 

In addition to HYALs, cell surface hyaluronidase transmembrane protein II (TMEM2, also known as CEMIP2) has recently been identified [21]. TMEM2 provides for the degradation of extracellular HMW HA into approximately 5 kDa fragments in a Ca^2+^-dependent manner at a pH optimum of between pH 6 and 7 [21]. TMEM2 comprises the predominant mediator of HA degradation and integrin-mediated cell substrate adhesions in cancer cells [22]. 

Cell Migration Inducing Hyaluronan Binding Protein (CEMIP), also known as KIAA1199 or HYBID (Hyaluronan-Binding Protein Involved In Hyaluronan Depolymerization), plays a role in HA binding and depolymerization in skin fibroblasts via clathrin endocytosis [23,24]. An acidic environment, H^+^ATPase, and dynamin are required for degradation mediated by CEMIP, resulting in 10–100 kDa HA [25]. It remains unclear whether CEMIP itself displays enzymatic activity; its role in the endocytosis-coupling process has proved difficult to verify. Selected properties of HYALs and HA-degrading proteins are summarized in Table 1.

Previous studies have provided somewhat controversial results regarding hyaluronidase activity in healthy human skin, mainly due to the application of differing methods, assay pH and models, including various human cell lines [13], reconstructed human epidermis (RHE) [26] and animal skin models [27]. Forming an understanding of HA degradation in the skin is crucial in terms of ensuring the long-term effectiveness of cosmetic products and dermal fillers [28]. In addition, the impaired homeostasis of HA in the ECM is able to significantly affect skin hydration. Interestingly, the localized digestion of HA in the ECM of the dermis may influence tissue inflammation and host antimicrobial defense [29].

The research thus comprised the characterization of the in situ distribution and the gene and protein expression of known HYALs, including in terms of their contribution to the degradation of HA in healthy human full-thickness skin ex vivo. We also expanded the established knowledge of the catabolism of HA in the skin by investigating recently identified TMEM2, as well as CEMIP proteins, accompanied by the assessment of their distribution and contribution to the depolymerization of HA. 

## 2. Materials and Methods

### 2.1. Biological Samples 

Human skin was obtained in the form of materials discarded following elective plastic eyelid surgery at the Galen surgical clinic (Ústí nad Orlicí, Czech Republic). The donors (women aged 45–75 years) provided their written informed consent for the use of the waste skin in the study. No additional risk or discomfort was suffered by the donors as a result of the use of the waste tissue. Porcine skin was obtained from auricles provided by a local slaughterhouse (Bocus Letohrad, Czech Republic).

### 2.2. Chemicals 

The following chemicals were purchased from Merck Life Science UK Ltd. (Dorset, UK): Acetic acid, Acrylamide, Agarose, Aluminum sulfate, Ammonium persulfate, Bovine Serum Albumin, D-glucose monohydrate, Dimethylaminobenzaldehyde, Dispase I solution, Ethylenediaminetetraacetic acid (EDTA), Fetal bovine serum, Formic acid, Hydrogen peroxide, Hydrochloric acid, l-glutamine Methylenebisacrylamide, Mowiol, *N*-acetyl-d-glucosamine, Paraformaldehyde, Potassium chloride, Potassium phosphate, Protease from *Streptomyces griseus* (Actinase E), Sodium dodecyl sulfate, Sodium hydroxide, Sodium chloride, Sodium phosphate, Sodium tetraborate, Sucrose, Stains-All solution, *Streptomyces hyaluronidase* (H1136), Tetramethylethylenediamine, Tris (Trizma base), Tween 20. Lach-Ner s.r.o. (Neratovice, Czech Republic) provided the Calcium chloride, Chloroform, Glycerol, Methanol, Sodium acetate, Triton X 100, Xylene. Bromophenol blue was obtained from Bio Basic (Markham, ON, Canada). BioTech (Minneapolis, MN, USA) provided Coomassie Blue, Glycine. Pertex was purchased from Histolab AB (Askim, Sweden), while ethanol 96%, and Hematoxylin Gill II were obtained from Bamed (Litvínovice, Czech Republic). P-LAB a.s. (Prague, Czech Republic) provided Alcian blue 8GX. Merck Millipore (Burlington, MA, USA) provided bHABP (biotinylated hyaluronan binding protein, 385911) and Nuclear Fast Red. ProLong Antifade Mountant with DAPI (4′,6-diamidino-2-phenylindole, P36962) was obtained from Invitrogen (Waltham, MA, USA). Processive HA lyase from *Streptococccus pneumoniae* (SpHyl, [30]), HMW and LMW HA and HA oligosaccharides were manufactured by Contipro a.s. (Dolní Dobrouč, Czech Republic).

### 2.3. Antibodies 

The following antibodies were used in the study: Anti-ACTB (Actin-β, sc-47778, Santa Cruz Biotechnology, Santa Cruz, CA, USA), anti-GAPDH (Glyceraldehyde-3-Phosphate Dehydrogenase, WH0002597M1, Merck Life Science UK Ltd.) anti-HYAL1 (bs-1235R, Bioss, Boston, MA, USA), anti-HYAL2 (bs-5888R, Bioss), anti-CEMIP (SAB2105467, Merck Life Science UK Ltd.), anti-TMEM2 (PA5-85901, Thermo Fisher Scientific, Waltham, MA, USA), Streptavidin-FITC (Fluorescein Isothiocyanate Conjugate, S3762, Merck Life Science UK Ltd.), Streptavidin-Alexa Fluor 647 (S21374, Invitrogen), goat anti-rabbit IgG AlexaFluor647 (A-21245, Thermo Fisher Scientific), goat anti-rabbit IgG AlexaFluor555 (ab150078), IgG (10500C Rabbit IgG Isotype Control, Thermo Fisher Scientific), Hoechst 34580 (H21486, Thermo Fisher Scientific).

### 2.4. Histochemical Staining for HA 

The histolocalization of the HA was determined using biotinylated bovine nasal cartilage-derived hyaluronan binding protein (bHABP) according to a technique described previously [31]. Briefly, the full-thickness skin was fixed in 4% PFA with acetic acid (5%) and ethanol (70%) for 24 h. Acidification and the addition of ethanol to the fixative were applied [32] in order to prevent the loss of HA due to its solubility in aqueous fixatives. This was followed by the standard deparaffinization and rehydration procedure. Subsequently, unspecific antibody binding was blocked in a blocking buffer containing 10% FBS for 30 min at RT. The samples were then stained with bHABP (dilution 1:50) overnight at 4 °C, followed by incubation with Streptavidin-AlexaFluor647 (dilution 1:100) for 30 min at RT and Hoechst 34580 (dilution 1:1000) for 10 min at RT and mounted in Mowiol. The images were taken using a TCS SP8 X (Leica, Wetzlar, Germany) confocal microscope with a 20× water immersion objective lens (HC PL APO CS2, 0.75 NA, Leica). The images were acquired in the form of z-stacks that covered the whole thickness of the slice with an increment size of 4 µm. The maximum projection operation was subsequently performed on a fluorescence channel z-stack series [33].

In order to verify the specificity of the HA staining, *Streptomyces hyaluronidase* treatment was performed prior to staining with bHABP. The sections were incubated with a solution of hyaluronidase from *Streptomyces hyalurolyticus*, 1 mg/mL in acetate buffer pH 5.4 (10 mM sodium acetate, 20 mM NaCl) for 1 h at 37 °C [34]. 

### 2.5. Homogenates from Human Skin, the Epidermis and Dermis 

Following excision, the skin samples were immediately immersed in physiological saline. Two tissue-weight volumes of physiological saline were added to the specimens, which were then disrupted using a homogenizer (TissueLyser II, Qiagen, Düsseldorf, Germany) with precooled sample holder cartridges. The homogenates were centrifuged at 18,000 *g* at 4 °C for 30 min, and the supernatants were collected. With respect to the dermis and epidermis, the human skin was incubated in 5 mL of Dispase I solution (2.4 U/mL) for 18 h, following which the epidermis and dermis were separated and the homogenates prepared accordingly. The skin homogenates were diluted with 0.15 M NaCl to a concentration of 10 mg/mL protein.

### 2.6. Quantification of the HA in the Lysate from the Skin Using Liquid Chromatography with Mass Spectrometric Detection (LC-MS/MS)

The HA in the skin homogenates was quantified as described by Šimek et al., 2019 [35]. Briefly, 100 µL of skin homogenate was digested with actinase E so as to disrupt the protein–HA interactions. The HA was then digested to unsaturated HA disaccharides with an HA lyase from *Streptococcus pneumoniae*; the disaccharides were subsequently quantified by means of LC-MS/MS. The chromatography separation was carried out using a Phenomenex Jupiter Proteo 4 µm 90 Å column (250 × 4.6 mm) employing the Acquity UPLC I-class chromatographic system (Waters, UK). Separation was performed at laboratory temperature via gradient elution with 0.1% formic acid in water and methanol. The flow rate was set at 0.5 mL/min, and the following methanol gradient was applied: 0–15% in 3–15 min, 15–80% in 15–16 min. The MS was performed using a triple quadrupole mass spectrometer equipped with an electrospray ion source (Xevo TQ-XS, Waters, UK) and operating in the negative ionization mode. Two characteristic product ions were monitored for both compounds.

### 2.7. LC-MS/MS Quantification of the HA Tetrasaccharides and Hexasaccharides

The quantification of HA tetrasaccharides and hexasaccharides in the skin lysates was performed using the same system as for the quantification of the HA. Prior to the analysis, a sample aliquot (10 µL) was diluted with 90 µL of water with the addition of an internal standard (^13^C-labeled tetra- and hexasaccharides, 50 µg/mL, 10 µL). The system was calibrated with the oligosaccharide standards using a 10-point calibration curve from 0.1–10 µg/mL. The chromatographic separation was performed on a Jupiter Proteo 4 µm 90 Å column (50 × 2.0 mm, Phenomenex, Torrance, CA, USA) at 60 °C. The column was eluted with a pure 0.1% formic acid solution in water for 3 min followed by a linear gradient of acetonitrile (0–30 %) over 7 min. The flow rate was set at 0.3 mL/min for an initial 2 min period and, after 0.5 min, it was increased to 0.6 mL/min. The column was washed following each run with 40% acetonitrile at 1 mL/min for 5 min and equilibrated with 100% 0.1% formic acid for 4 min. The injection volume was set at 5 µL. Two characteristic product ions were acquired for each compound.

### 2.8. Colorimetric Hyaluronidase Activity Assay 

The activity of the hyaluronidases in the skin homogenates was assessed as previously described by Morgan and Elson [36]. Briefly, the homogenate was incubated with exogenous HA HMW (1.5 MDa) for 24 h at 37 °C. Three incubation reactions with differing pH values were prepared (pH 3.5, pH 5.4 and pH 7) in the absence or presence of 1 mM calcium chloride. The reactions were then heat inactivated (100 °C for 5 min). The number of HA ends was quantified via the Morgan–Elson color reaction (potassium tetrahydroborate and acidified dimethylaminobenzaldehyde), and the absorbance was measured at 585 nm. 

The concentration of GlcNAc-reducing ends in each sample was interpolated from a concurrently prepared GlcNAc standard. With respect to the HA size analysis via agarose gel electrophoresis, HMW HA (1.5 MDa) was incubated with the homogenates from the whole human skin, epidermis, dermis and porcine skin at 37 °C for 96 h. The HA was stained blue on the agarose gel using Stains-All. Low molecular weight (LMW; 15 kDa) and HMW HA served as the controls. 

### 2.9. Substrate Gel Electrophoresis—Zymography Assay 

The zymographic detection of hyaluronidase activity was performed as described previously [37,38]. In brief, the samples (skin homogenates) were electrophoresed through 1% SDS-PAGE containing 0.4 mg/mL of exogenous HMW HA (1.5 MDa). Following electrophoresis, the gels were washed in 2.5% Triton-X and subsequently incubated in developing buffer (0.15 M NaCl; 0, 1 M HCOOH; three developing buffers with distinct pH values were prepared—pH 3.5, pH 5.4 and pH 7, and in the absence or presence of 1 mM CaCl_2_) at 37 °C for 18 h. The gels were then stained with 0.5% Alcian blue in 3% acetic acid, via which the hyaluronidase activity was visualized as clear bands on the blue background.

### 2.10. HA Size Analysis Via Agarose Gel Electrophoresis 

The hyaluronidase activity in the homogenates from human and porcine skin was determined via incubation with exogenous 1.5 MDa HMW HA. Briefly, the homogenates were incubated with HMW HA in reaction with distinct pH values (pH 3.5, pH 5.4 and pH 7.0) in the absence or presence of 1 mM Calcium chloride for 24 h at 37 °C. The samples and the standards of HA (size 20 kDa; 1.5 MDa) were then electrophoresed through a 0.5% agarose gel in a TAE buffer (40 mM Tris, 20 mM Acetic acid and 1 mM EDTA, pH 8.3) for 1 h at a constant current of 100 V. The HA was visualized via staining in 0.005% Stains-All overnight in the dark and de-staining under normal light in water until distinct bands became visible.

### 2.11. Determination of the Protein Concentration in the Skin Homogenate 

The protein content was determined using a Thermo Fisher Scientific Pierce BCA Protein Assay Kit (Thermo Fisher Scientific) according to the manufacturer’s instructions. The results were obtained in units of mg/mL, subsequently re-calculated to the amount of tissue. The results were then presented in units of mg/g of skin tissue (for the protein concentration). Two independent experiments were performed; data from both are presented.

### 2.12. Gene Expression Quantification with the Quantitative Real-Time Polymerase Chain Reaction (RT-qPCR) 

The total RNA from the human full-profile skin, dermis and epidermis (the dermis and epidermis were prepared as described in the preparation of the lysate from human skin) was isolated using the RNeasy kit (Qiagen), and the cDNA synthesis was performed using the SuperScript III First Strand Synthesis System (Thermo Fisher Scientific). cDNA was used as a template in TaqMan real-time PCR assays (StepOne™ Real-Time PCR System; ThermoFisher Scientific) according to the manufacturer’s instructions. The TaqMan assays used for the RT-qPCR comprised: ACTB Hs01060665_g1, HYAL1 Hs00201046_m1, HYAL2 Hs00186841_m1, HYAL3 Hs00185910_m1, HYAL4 Hs00202177_m1, HYAL5 Hs00162139_m1, CEMIP Hs01552114_m1 and TMEM2 Hs00910521_m1 (Thermo Fisher Scientific). The relative quantification threshold values (Ct) of the HYALs, TMEM2 and CEMIP were normalized by subtracting the Ct of the housekeeping gene β-actin (ACTB) so as to obtain _Δ_Ct. The relative expression was then expressed as 2^-ΔCt^. This transformation allowed for the comparison of the relative gene expression levels for the respective gene and reflected the “signal strength” of the respective assays. 

### 2.13. Western Blot 

Western blot analysis was performed for the homogenates from the normal human skin. The homogenates were separated by applying 10% SDS-PAGE and transferred to polyvinylidene difluoride membranes. The blots were incubated with the following primary antibodies: anti-ACTB, anti-GAPDH, anti-HYAL1, anti-HYAL2, anti-CEMIP and anti-TMEM2 (1:500 dilution in PBS, pH 7.5, 5% dried milk powder, 0.1% Tween 20). Anti-GAPDH and anti-ACTB were used as the loading controls. The blots were developed with peroxidase-conjugated anti-rabbit or anti-mouse IgG applying chemiluminescent detection, so as to reveal and visualize the Western blot signals (SuperSignal West Femto Max, 34095, Thermo Fisher Scientific). The membranes were exposed and scanned using an Alliance 9.7 Chroma Chemiluminescence Imaging System (UVItec Ltd., Cambridge, UK).

### 2.14. Immunohistochemistry, Immunohistofluorescence 

The immunohistochemistry (IHC) and immunohistofluorescence (IHF) were performed according to the manufacturer’s instructions (IHC Guidebook DAKO). Briefly, the IHC studies were performed on paraffin sections of formalin-fixed healthy human skin; the sections were deparaffinized and rehydrated. Antigen retrieval was performed in Tris HCl/EDTA buffer, pH 9 for 20 min at 95 °C. Unspecific antibody binding was blocked following permeabilization and the blocking of the endogenous peroxidase activity. The antigen was subsequently detected via incubation (1 h at 37 °C) with anti-rabbit primary antibodies: anti-IgG (Rabbit IgG Isotype Control), anti-HYAL1, anti-HYAL2, anti-CEMIP and anti-TMEM2 (1:100 dilution in blocking buffer—10% BSA in TBS, pH 7.5, 0.1% Tween 20). Regarding the IHC, the detection was performed via incubation with an anti-rabbit secondary antibody conjugated with peroxidase (30 min at 37 °C), followed by diaminobenzidine development (Envision kit; DAKO). The samples were then washed and counterstained (30 s) with Hematoxylin Gill II solution and mounted with Pertex resinous mounting medium. Concerning the IHF, the samples were incubated with goat anti-rabbit IgG (conjugated either with Alexa Fluor 647 or Alexa Fluor 555) at 1:500 dilution for 1 h at RT, followed by Hoechst staining (dilution 1:500) for 10 min at RT and mounting into Mowiol. Images were taken using a Nikon Eclipse 50i microscope with an integrated Nikon DS Fi1 camera and a Leica TCS SP8 confocal microscope.

## 3. Results

### 3.1. Hyaluronan Distribution in the ECM of Healthy Human Skin 

Its lack of immunogenicity comprises one of the most significant assets of HA; it acts to prevent the production of specific antibodies against HA [34,39]. Therefore, immunohistochemical methods are of no use in the detection of HA. We studied the distribution of HA in healthy human and porcine skin in the form of histological tissue sections using HA-specific binding peptide (bHABP) derived from bovine nasal cartilage [40]. bHABP has previously been used to identify HA in skin tissue sections [41]; however, in this case, the HA was observed using a light microscope, which resulted in insufficient appreciation of the structure of HA network. Therefore, we used a confocal microscope, which allowed for the three-dimensional reconstruction of the spatial distribution of the HA in the skin tissues.

**Figure 1:**
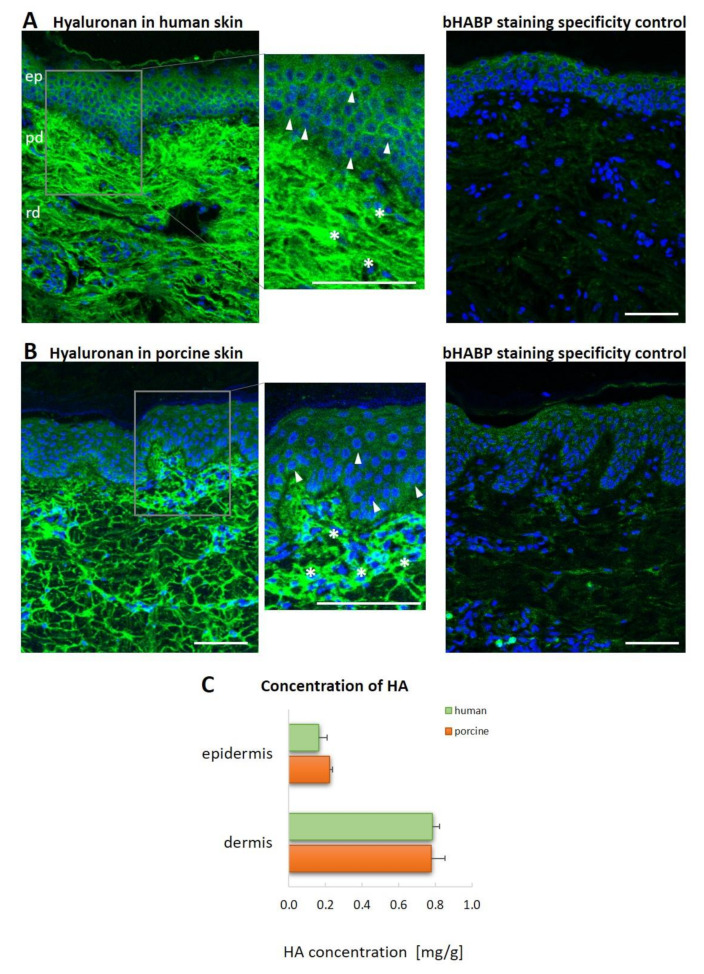
Patterns of hyaluronan staining and the staining concentration in healthy human and porcine skin. (**A**,**B**) The histofluorescent staining of HA (green) with bHABP in normal (**A**) human and (**B**) porcine skin tissues, fixed and paraffin embedded. Hyaluronidase from *Streptomyces hyalurolyticus* was used as the control for the bHABP staining specificity. The localization of HA was visualized using a streptavidin-conjugated Alexa 647 and a confocal microscope. The image represents the maximum projection of the z-stack sections. The insets show enlarged views of the regions in the gray boxes. The nuclei were stained with Höechst (blue). The asterisks indicate fibroblasts, and the arrows indicate keratinocytes. The scale bar at the bottom of the figure represents 100 μm. *n* = 3 independent experiments. Abbreviations: biotinylated hyaluronan binding protein (bHABP), epidermis (ep), papillary dermis (pd), reticular dermis (rd). (**C**) Quantification of HA in the human and porcine skin. HA content in 100 µL of lysate from the epidermis and dermis was quantified via LC-MS/MS; the results were then adjusted for the initial tissue weight. The results are presented in units of mg/g of skin tissue. The gray bars represent the means of *n* = 3 independent biological replicates with SEM.

While some cells in the epidermis and dermis stained positive for HA, the signal was observed predominantly in the ECM of both human and porcine skin (Figure 1A,B). The epidermis demonstrated weaker HA staining. The HA in the dermis stained more intensely and formed a web-like structure. The most intense HA staining was observed in the papillary dermis, with weaker staining in the reticular dermis. The localization of HA appeared to be comparable for the two species (Figure 1A,B). The specificity of the bHABP staining was evaluated via the treatment of skin sections with *Streptomyces hyaluronidase* prior to histological staining. As a result, a significantly decreased signal was detected following the hyaluronidase treatment, thus confirming the specificity of the bHABP (Figure 1A,B). 

Aimed at the assessment of the HA content, analytical techniques (LC-MS/MS) were employed so as to determine the concentration of HA in the homogenates prepared from the epidermis and dermis. The HA concentration was found to be almost the same in the human and the porcine skin. The amount of HA was approximately five times higher in the dermis than in the epidermis for both species (Figure 1C). 

### 3.2. Hyaluronidases Are Expressed Differentially in the Human Epidermis and the Dermis

**Figure 2:**
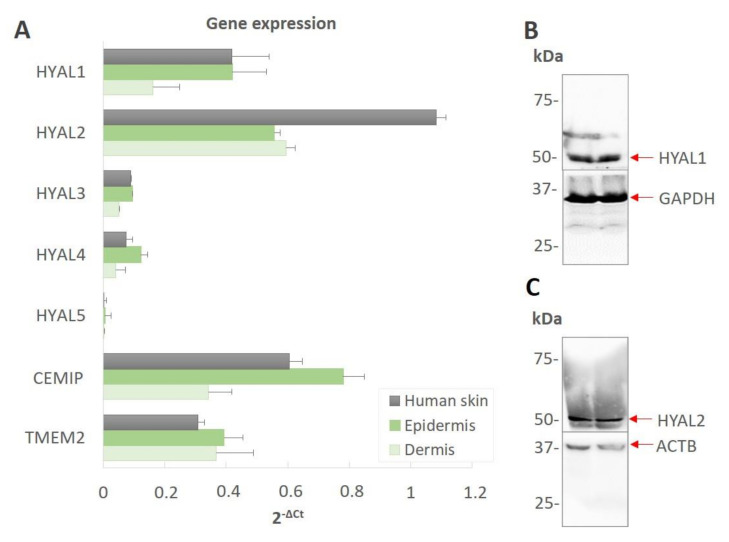
The expression of HYALs, CEMIP and TMEM2 in the human skin and the detection of proteins via Western blotting. (**A**) The relative expression levels of the HYAL1, HYAL2, HYAL3, HYAL4, HYAL5, CEMIP and TMEM2 transcripts were measured using RT-qPCR. The average values from three independent experiments are shown. The error bars represent the SEM. (**B**) Western blot analyses of the human skin lysates (in duplicates) with anti-HYAL1 and anti-GAPDH antibodies and (**C**) anti-HYAL2 and anti β-actin antibodies. GAPDH and ACTB were used as the loading controls. Abbreviations: Hyaluronidase (HYAL), Transmembrane Protein type II (TMEM2), Cell Migration Inducing Hyaluronan Binding Protein (CEMIP), Glyceraldehyde 3-phosphate dehydrogenase (GAPDH), Actin Beta (ACTB).

**Figure 3:**
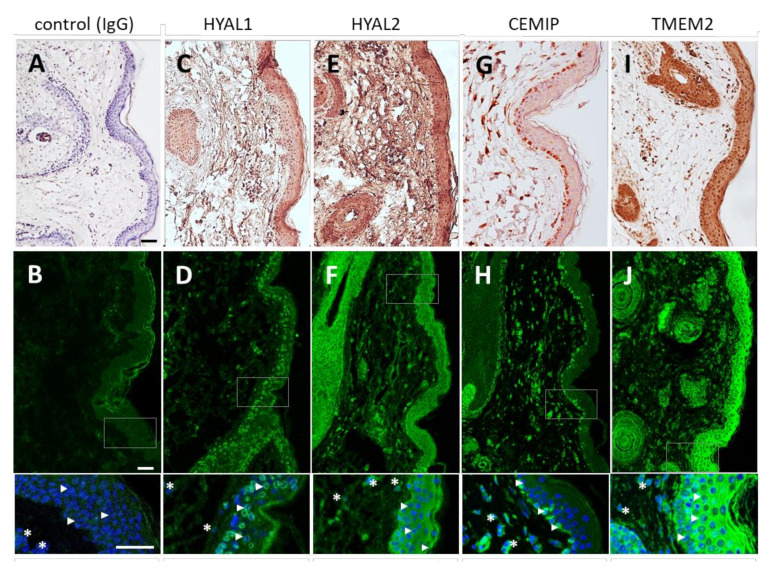
Localization of HYAL1, HYAL2, CEMIP and TMEM2 in the human skin. (**A**,**B**) Immunohistochemical and immunohistofluorescent analysis of IgG, (**C**,**D**) HYAL1, (**E**,**F**) HYAL2, (**G**,**H**) CEMIP and (**I**,**J**) TMEM2. The insets provide enlarged views of the regions in the gray boxes. The nuclei were stained with Höechst (blue). The asterisks indicate the fibroblasts, and the arrows indicate the keratinocytes. The scale bars at the bottom of the figures represent 50 μm. Abbreviations: hyaluronidase (HYAL), Transmembrane Protein type II (TMEM2), Cell Migration Inducing Hyaluronan Binding Protein (CEMIP).

The gene expression of HYALs, CEMIP and TMEM2 was assessed via qRT-PCR in the human full-thickness skin, epidermis and dermis. As demonstrated in Figure 2, each of the three experimental groups expressed HYAL1, HYAL2, CEMIP and TMEM2. The expression of HYAL3 and HYAL4 in the human skin was relatively low. No HYAL5 mRNA was detected (Figure 2A). Because of the low gene expression and the lack of published reports of the enzymatic activities of HYAL3 (C_t_ 33), HYAL4 (C_t_ 34) and HYAL5 (C_t_ 39) in skin [19,42], we validated only the HYAL1 and -2 via Western blotting. The skin samples were found to contain both HYAL1 and HYAL2 (Figure 2B,C). HYAL1 was detected in two bands, the smaller of which corresponded to approximately 50 kDa. HYAL2 migrated as a single band at around 50 kDa. The two HYAL1 bands may represent multiple isoforms [43]. Thus, the data obtained from the WB analysis indicate that both enzymes (HYAL1 and HYAL2) are present in human skin. The WB analysis was further applied in the identification of the presence of CEMIP and TMEM2 in the human skin lysate. Neither CEMIP nor TMEM2 were successfully detected in the skin (data not shown) due to the lack of validated antibodies for the application of Western blotting. In addition, HYAL1, HYAL2, CEMIP and TMEM2 were localized in the epidermis and dermis at the protein level via the IHF and IHC (Figure 3). 

The high-quality histological observations (Figure 3) of the healthy human skin showed that HYAL1 was highly expressed in the epidermis, especially in the stratum granulosum and stratum spinosum. At the same time, only a small number of cells stained positive in the dermis, which correlated with the gene expression pattern. HYAL1 localized most probably to lysosomes (Figure 3C,D). HYAL2 was detected in both the epidermis and the dermis. Concerning the epidermis, the HYAL2 staining was mostly extracellular (Figure 3E,F). In comparison, the HYAL2 stained in the dermis both extra- and intra-cellularly; the HYAL2 staining was diffuse in the cytoplasm. CEMIP was found to be intracellular in both the epidermal keratinocytes and the dermal fibroblasts. Interestingly, the epidermal localization of CEMIP was restricted to the basal layer (Figure 3G,H). For the first time, the exact localization of TMEM2 was immunodetected in the epidermis and dermis. TMEM2 protein was found to be abundant in the human skin; its localization was similar to that of HYAL2. Concerning the epidermis, the TMEM2 signal was prominent intracellularly in all the epidermal layers. Thanks to the precise and detailed photography, TMEM2 was found to be diffuse in the cells (except for the nuclei) and was not detected at the cell interface. TMEM2 was localized in the dermis intracellularly in the fibroblasts (Figure 3I,J). Since HYAL1, HYAL2, CEMIP and TMEM2 were detected at a gene expression level that correlated with the IHC and IHF staining, these proteins can participate in the turnover of HA in the skin.

### 3.3. Skin Homogenates Contain Components with Hyaluronidase Activity 

**Figure 4:**
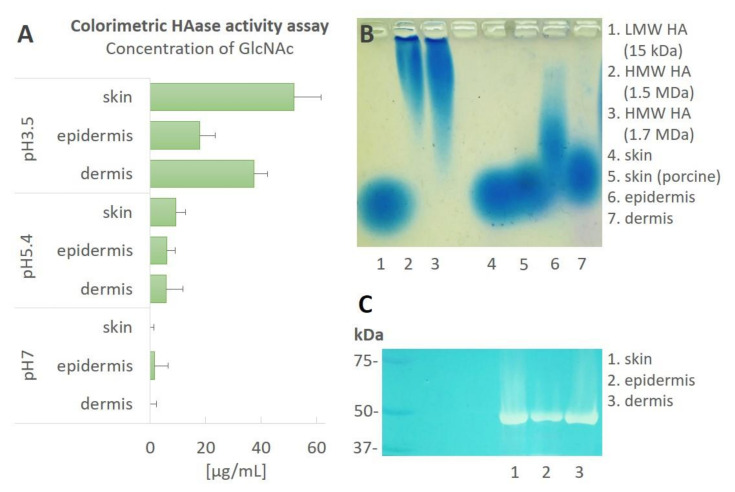
Hyaluronidase activity in the skin. Hyaluronidase activity was analyzed in the homogenates (10 mg proteins/mL) from the human skin, epidermis and dermis. (**A**) Colorimetric hyaluronidase activity assays of the samples incubated at differing pH values (pH 3.5, pH 5.4 and pH 7). The values represent the means of three independent biological samples ± the SEM of the GlcNAc concentrations. (**B**) HA size analysis via agarose gel electrophoresis at pH 3.5. The samples were incubated with exogenous HMW HA (1.5 MDa) at 37 °C for 24 h. The samples were then analyzed via agarose gel electrophoresis and HA staining (blue). The LMW HA and both HMW HA served as the size references. (**C**) A representative zymography using HMW HA as a substrate. The left-most lane shows a protein ladder. Abbreviations: low molecular hyaluronan (LMW HA), high molecular hyaluronan (HMW HA), N-acetylglucosamine (GlcNAc).

The hyaluronidase activity was studied in the homogenates of the human full-thickness skin and the separated epidermis and dermis. Since hyaluronidases exhibit varying pH optima, the experiments were performed at differing pH values (3.5, 5.4 and 7) and in the presence or absence of CaCl_2_. The activity of the hyaluronidases at pH 5.4 and pH 7 was either very low or undetectable (Figure 4A). Moreover, HYAL1 was observed to be most active at a pH of 3.5 (Figure S1D). The presence of CaCl_2_ did not influence the activity of the hyaluronidase (Figure S1A; data from the colorimetric hyaluronidase activity assay and zymography are not shown). Therefore, Figure 4B,C illustrate the experiments performed at a pH of 3.5 and in the absence of CaCl_2_.

Hyaluronidase activity was detected in the human skin, epidermis and dermis (Figure 4 and Figure S1). The colorimetric hyaluronidase activity results suggested that the enzymatic activity exerted by the tissue homogenates can be attributed to HYAL1 or HYAL2, which evince optimal activities at acidic pH values and are (unlike TMEM2) independent of the presence of CaCl_2_ (Figure 4A). We have also observed similar HA degrading activity at low pH in lysates from human and porcine skin samples (Figure S1B). The cleavage of HA by the skin enzymes was further characterized by applying HMW HA as a substrate. The HMW HA (1.5 MDa) cleaved to the LMW HA primarily (around 20 kDa) via the lysate of the full-thickness skin. With regard to the lysate from the epidermis, the HA fragments were larger than 20 kDa, which suggested that the hyaluronidases in the epidermis exhibited lower activity and, thus, did not completely cleave the HMW HA (Figure 4B). The HA substrate gel zymography at acidic pH values revealed a single band of activity in the tissue homogenates (Figure 4C); the band corresponded approximately to 50 kDa. The size of the band suggested that the HA-degrading enzyme(s) active in the skin lysates were either HYAL1 (size of approximately 50 kDa) or HYAL2 (size of approximately 54 kDa), thus corresponding to the Western blot results (Figure 2B,C). 

Aimed at distinguishing between the HYAL1 and HYAL2 activities in the lysates, we analyzed the concentrations of HA tetrasaccharides (HA4AN) and hexasaccharides (HA6AN) in the homogenates incubated with HMW HA. The amount of these small fragments was low, suggesting that HYAL2 is responsible for the degradation of HMW HA in skin tissues (Figure S1C). 

## 4. Discussion

### 4.1. HA in Human Skin Is Degraded by HYAL1 and HYAL2

HYALs are found in the human body in relatively low concentrations and are difficult to purify, characterize and measure with respect to their high activity due to their extreme instability [17,44]. Hence, HYALs have received limited attention to date, and very little information is available in the literature on their function and localization in the skin. However, new procedures and techniques now enable the isolation and characterization of HYALs [45]. This study aimed at localizing HYALs and at identifying both the hyaluronidase activity and the direct involvement of particular HYALs in normal human full-thickness skin. In this respect, the availability of commercially produced antibodies facilitated the execution of research, and the additional IHC, IHF, WB and zymography data supported the above observation. The anti-HYAL1 antibody revealed that HYAL1 is localized (IHF) intracellularly in all the layers of the epidermis, particularly in the stratum spinosum and stratum granulosum, and less so in the basal layer and the dermis. This correlates with both the gene expression and information provided in the literature. While Malaisse et al. demonstrated the presence of HYAL1 in the epidermis, no detailed images were provided concerning its distribution in the epidermal layers [26]. Further, it has been found that HYAL1 localized into lysosomes in macrophages and liver homogenates [46,47], in line with which our data indicated similar lysosomal localization in keratinocytes. 

The mechanisms via which HA is cleaved in the ECM are not yet fully understood. The widely accepted model [48] proposes that HMW HA is tethered to the cell surface by an HA receptor cluster of differentiation (CD44) that is concentrated in the form of caveolin-rich lipid rafts [49]. HA is then cleaved by HYAL2 into intermediate-size fragments in acidic microenvironments created by an Na+/H+ exchanger that enables HYAL2 activity [16]. The intermediate-size fragments are then internalized and degraded to monosaccharides via the combined action of lysosomal hyaluronidases and exoglycosidases (reviewed in [17]). Our data supported this model in several respects. (1) We determined that HYAL2 is localized in keratinocytes and fibroblasts, both intra- and extracellularly, and that HYAL1 is localized intracellularly, most likely in the lysosomes. (2) Although the degrading activity of HYAL2 HA is at least 50 times lower than that of HYAL1, we found that HYAL2 is present in the human epidermis and dermis in relatively high concentrations. Such amounts of HYAL2 are thought to be sufficient to degrade the HMW HA in the ECM. Moreover, the 24 h degradation of HMW HA resulted in the production of intermediate-size fragments of around 20 kDa (the end products of HYAL2) rather than hexa- and tetrasaccharides (the end products of HYAL1). (3) We discovered that HYALs in the skin are acid active only (a pH of 4 is optimal for HYAL1 and HYAL2). A further form of HYAL2 was found to be soluble and was detected in the lysosomes with HYAL1 (reviewed in [9]), together with which it assists in the degradation of HA. 

Thus, we concluded that HYAL2 comprises the main HA-degrading enzyme in the skin (based on the size of HA fragments). Our data were partially supported by a recent study that reported that both enzymes (HYAL1 and HYAL2) are involved in HA catabolism in the skin, since HA accumulates within these tissues in both types of knockout mice. The mean increases in the amount of HA in the skin were found to be 2- and 4-fold in HYAL1 and HYAL2 knockout mice, respectively, compared to wild-type mice. However, a further HA-degrading protein, most probably a combination of HYAL1, CEMIP and TMEM2, is required for the degradation of HA to less than 20 kDa fragments and a turnover of HA in the skin.

### 4.2. The Involvement of Other Proteins in the Degradation of Cutaneous HA

Recent studies have verified the existence of a newly identified HA-degrading enzyme in mouse organs—TMEM2, a hyaluronidase anchored to the plasma membrane [21]. TMEM2 is a hyaluronidase that (1) localizes to the cell surface, (2) eliminates substrate-bound HA in the cell-substrate contact area and (3) has a pH optimum of pH 6–7 [21]. However, these findings were not fully supported by our data. We were unable to detect any hyaluronidase activity in the homogenate from the human skin at pH 7—the published optimal pH for TMEM2; even the presence of Ca^2+^ exerted no effect on hyaluronidase activity. We detected a relatively high TMEM2 gene expression and, for the first time, a TMEM2 protein signal localized in both the dermis and the epidermis. In addition, we demonstrated the diffuse localization of TMEM2 in epidermal cells. The expression of TMEM2 mRNA in human skin and its localization in the skin appears to suggest the important role of TMEM2. The data indicated three possible explanations: (1) TMEM2 is not active in the human skin; (2) TMEM2 may have a very low activity, which we were unable to detect under our laboratory conditions; or (3) TMEM2 has a relatively high activity, which was disrupted during extraction. Our data are supported by a recent study that showed that, although normal human skin fibroblasts express TMEM2, the knockdown of TMEM2 by siRNA does not act to abrogate HA degradation. Therefore, little evidence is available of the direct involvement of TMEM2 in the degradation of HA in human cells, such as skin fibroblasts [23] (reviewed in [24]). Thus, based on our data and data from Yoshino et al. 2018, we concluded that despite the presence of the TMEM2 mRNA and the corresponding protein in human skin, TMEM2 does not appear to participate in the catabolism of cutaneous HA. 

The participation of the CEMIP protein in the degradation of HA was recently revealed [25]. In a similar way to TMEM2, CEMIP is an HA-degrading protein that is thought to be active in human skin. Although CEMIP bears structural similarities to TMEM2 [21], it has a number of features that are inconsistent with the presumption that it comprises an active skin hyaluronidase. For example, although CEMIP is secreted extracellularly, even the culture supernatants of CEMIP-overexpressing cells demonstrated very little HA degrading activity. CEMIP-mediated HA degradation requires the participation of a clathrin-coated pit pathway, thus suggesting that CEMIP may function in the intracellular compartments and is detected primarily intracellularly, which correlates with our results. Thus, despite their structural similarities, the properties of TMEM2 and CEMIP are distinct [23,27]. 

Our data showed that the CEMIP mRNA and protein are present intracellularly in the dermis and, for the first time, in the epidermis. Concerning the epidermis, CEMIP is localized exclusively to basal keratinocytes in which it potentially participates in the turnover of HA; a possible explanation concerns the need for a higher level of HA turnover in the basal layer of proliferating active cells. Nevertheless, the activity of CEMIP in the human epidermis and dermis was not studied in our research. Although the way in which CEMIP contributes to the degradation of HA in the skin remains unknown, its expression and protein localization in the skin suggest that CEMIP is likely to be involved. Alternatively, CEMIP may function as an adaptor molecule for an unknown hyaluronidase via which it assists in the internalization of intermediate-sized HA into the cell. A further explanation suggests that CEMIP induces the degradation of HA when bound to the ANXA1 protein [50]. Interestingly, a recent report by Dokoshi et al. indicated that CEMIP is the major inducible gene responsible for HA catabolism in mice. CEMIP−/− mice failed to digest HA [29]. A further study revealed that CEMIP appears to play an essential role in human skin, since the knockdown of CEMIP in NHDF fibroblasts acted to eliminate the degradation of HA [51].

### 4.3. HA Homeostasis Differs between the Epidermis and Dermis

Our findings in the content of HA in both human and porcine skin revealed that HA is localized significantly more in the dermis than in the epidermis. Porcine skin contains a similar amount of HA as human skin, and its distribution pattern is also similar, which suggests that this is a universal pattern that is not dependent on differing skin types, the localization of the skin or sex. Our results corresponded with previous findings [10] that demonstrated that HA occurs in both the epidermis and dermis, with the dermis containing a higher amount of HA. HA is synthesized as a high molecular mass compound, both in the epidermis and the dermis [10]. Although the amount of newly synthesized HA is, on average, significant in both skin layers, the epidermis contains more HMW HA. The degradation of epidermal HA is slightly faster than that of the HA in the dermis. Therefore, it is thought that the metabolic routes of epidermal and dermal HA differ [10]. Our data showed that the catabolism of HA in the dermis was more intense than in the epidermis (Figure 4A).

With respect to its turnover, HA is removed from the dermis via both diffusion followed by systemic degradation and in situ degradation (avascular) [13], while HA degradation occurs exclusively in situ in the epidermis. HMW HA is entrapped within the ECM between the keratinocytes and is limited by the *stratum corneum* above and the basal lamina below [52]. Our results that indicated that the expression of HYAL1 in the epidermis is higher than in the dermis are consistent with the fact that the catabolism of epidermal HA takes place exclusively in situ, while dermal HA is partly drained via the lymphatics. Extracellular HMW HA is thought to be cleaved by HYAL2 in the epidermis (this takes place at an acidic pH caused locally by proton pumps), and keratinocytes internalize the resulting HA fragments, which are delivered to endosomes and lysosomes where HYAL1 catabolizes the fragments into small oligosaccharides [5]. This was supported by our IHF data—HYAL1 is localized intracellularly, whereas HYAL2 is extracellular. Based on our IHF and expression data, catabolism is thought to be supported by CEMIP in the *stratum basale* due to its exclusive expression in this single layer of the epidermis. The intracellular localization of CEMIP in the keratinocytes corresponds to in situ HA degradation. CEMIP also appears to be involved in the degradation of dermal HA, thus serving to confirm data published by Yoshida et al. 2013 that indicated the importance of the CEMIP protein for the catabolism of HA in fibroblasts [25]. According to the spatial distribution of HA-degrading proteins indicated by our confocal microscope photographs, HYAL2, CEMIP and TMEM2 share intracellular localization within the dermis.

**Figure 5:**
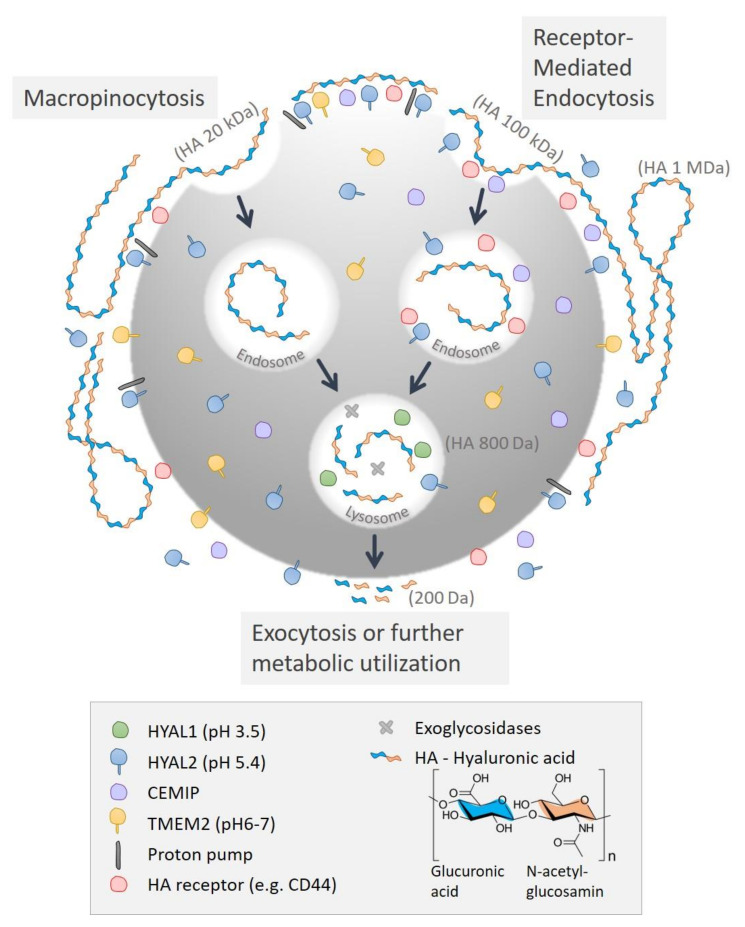
A suggested metabolic scheme of the degradation of hyaluronan and the localization of HA-degrading proteins in human skin. HMW HA is hydrolyzed by HYALs. This most probably initially occurs on the plasma membrane via HYAL2. The HA is then internalized into the cell via the use of surface HA scavenger receptors, CEMIP or a binding protein (e.g., CD44) for receptor-mediated endocytosis or macropinocytosis. Once internalized, the HA is degraded by HYAL1, HYAL2 and exoglycosidases in the lysosomes into small fragments and subsequently exocytosed. Modified from Csoka et al. (2001) [53] and Racine and Mummert (2012) [54]. According to our data, this mechanism is thought to occur mainly within dermal fibroblasts and basal keratinocytes. The upper keratinocytes appeared to lack the expression of CEMIP. Abbreviations: Hyaluronidase (HYAL), Transmembrane Protein type II (TMEM2), Cell Migration Inducing Hyaluronan Binding Protein (CEMIP), Cluster of differentiation 44 (CD44).

A suggested metabolic mechanism governing the degradation of HA and the localization of HA-degrading proteins in human skin is shown below in Figure 5.

The regulation of the turnover of HA in the epidermis and the dermis is a very complex process and the exact mechanism is not yet fully understood. A knowledge of the natural degradation of HA has the potential to contribute to the development of more stable and hyaluronidase-sensitive implants and HA-based treatments (such as dermal fillers). The precise localization of HA-degrading proteins is of particular importance. Finally, the contributions of the various HA-degrading proteins to the overall activity of hyaluronidase will require an execution of knockout experiments going forward.

## 5. Conclusions

In summary, this study demonstrated that HYAL2 is a key player at the level of human skin HA clearance pathways (the detection of 20 kDa fragments). No or very low hyaluronidase activity was exerted by the HYAL1 (tetrasaccharides not detected) and TMEM2 (no activity at a pH of 7 and independent of CaCl_2_). Despite the presence of TMEM2 and CEMIP in the skin, their roles in the degradation of HA in the skin remains unclear. The enhanced knowledge of the distribution of HYAL1, HYAL2, TMEM2 and CEMIP in full-thickness human skin provided by our study serves to enhance the overall understanding of the catabolism of HA and its functional significance in skin development and diseases. The study revealed for the first time that the localization of TMEM2 within cells is primarily intracellular in keratinocytes and fibroblasts. CEMIP was found to be intracellular in both dermal fibroblasts and, interestingly, in basal epidermal keratinocytes. The visualization of the location of HA in skin tissues together with that of HA-degrading proteins, and the confirmation of their activity in skin tissues, enhances present knowledge of the localization of HA and its metabolism in the skin. Thus, the elucidation of the localization of HA and its degradation in the skin opens up new avenues for the investigation of novel pharmacological targets aimed at, for example, confronting the age-related turnover of HA in the skin and introducing new innovative ideas to the fields of cosmetology, dermatology and immunology.

## Figures and Tables

**Table 1:** Overview of HA-degrading proteins.

HA-Degrading Proteins	Subcellular Localization/Tissue Distribution	Substrate	End Products of HA Degradation	pH Optimum
HYAL1	lysosomes/ubiquitous	HA of any size	tetrasaccharides (0.8 kDa)	3.5
HYAL2	cytoplasmic membranes, ECM/ubiquitous	HMW HA	oligomers (20 kDa)	4
HYAL3	intracellular/testicles, bone marrow, less other tissues	HA	no hyaluronidase activity	acidic
HYAL4	cytoplasmic membranes/placenta, skeletal muscle	chondroitin sulfate	-	-
PH20	cytoplasmic membranes/testicles	HA, chondroitin sulfate	tetrasaccharides	neutral, acidic
TMEM2 (CEMIP2)	cytoplasmic membranes/ubiquitous, placenta	HMW HA	5 kDa	6–7
CEMIP	cytoplasmic membranes, cytoplasm/lymph node, endometrium	HMW HA *	10–100 kDa	acidic

* It remains unclear whether CEMIP has direct catalytic activity as an enzyme. Abbreviations: hyaluronidase (HYAL), hyaluronidase PH-20 (PH20), Transmembrane Protein a type II (TMEM2), Cell Migration Inducing Hyaluronan Binding Protein (CEMIP), extracellular matrix (ECM), hyaluronan (HA), high molecular weight hyaluronan (HMW HA).

## Data Availability

Not applicable.

## Supplementary Materials

The following are available online at https://www.mdpi.com/article/10.3390/biom12020251/s1, Figure S1: Hyaluronidase activity in the skin.

## References

[1] Kogan G., Šoltés L., Stern R., Gemeiner P. (2007). Hyaluronic acid: A natural biopolymer with a broad range of biomedical and industrial applications. Biotechnol. Lett..

[2] Huerta-Angeles G., Nešporová K., Ambrozova G., Kubala L., Velebný V. (2018). An Effective Translation: The Development of Hyaluronan-Based Medical Products From the Physicochemical, and Preclinical Aspects. Front. Bioeng Biotechnol..

[3] Schnabelrauch M., Scharnweber D., Schiller J. (2013). Sulfated glycosaminoglycans as promising artificial extracellular matrix components to improve the regeneration of tissues. Curr. Med. Chem..

[4] Toole P.B., Wight T.N., Tammi M.I. (2002). Hyaluronan-cell interactions in cancer and vascular disease. J. Biol. Chem..

[5] Tammi I.M., Day A.J., Turley E.A. (2002). Hyaluronan and homeostasis: A balancing act. J. Biol. Chem..

[6] Muto J., Sayama K., Gallo R.L., Kimata K. (2019). Emerging evidence for the essential role of hyaluronan in cutaneous biology. J. Dermatol. Sci..

[7] Ganceviciene R., Liakou A.I., Theodoridis A., Makrantonaki E., Zouboulis C.C. (2012). Skin anti-aging strategies. Dermatoendocrinol.

[8] Laurent C.T., Fraser J.R. (1992). Hyaluronan. FASEB J..

[9] Lepperdinger G., Mullegger J., Kreil G. (2001). Hyal2—Less active, but more versatile?. Matrix Biol..

[10] Tammi R., Säämänen A.-M., I Maibach H., Tammi M. (1991). Degradation of newly synthesized high molecular mass hyaluronan in the epidermal and dermal compartments of human skin in organ culture. J. Investig. Derm..

[11] Erickson M., Stern R. (2012). Chain gangs: New aspects of hyaluronan metabolism. Biochem. Res. Int..

[12] Reed K.R., Lilja K., Laurent T.C. (1988). Hyaluronan in the rat with special reference to the skin. Acta Physiol. Scand.

[13] Stair-Nawy S., Csoka A.B., Stern R. (1999). Hyaluronidase expression in human skin fibroblasts. Biochem. Biophys. Res. Commun..

[14] Afify A.M., Stern M., Guntenhoner M., Stern R. (1993). Purification and characterization of human serum hyaluronidase. Arch. Biochem. Biophys..

[15] Lepperdinger G., Strobl B., Kreil G. (1998). HYAL2, a human gene expressed in many cells, encodes a lysosomal hyaluronidase with a novel type of specificity. J. Biol. Chem..

[16] Bourguignon L.Y., Singleton P.A., Diedrich F., Stern R., Gilad E. (2004). CD44 interaction with Na+-H+ exchanger (NHE1) creates acidic microenvironments leading to hyaluronidase-2 and cathepsin B activation and breast tumor cell invasion. J. Biol. Chem..

[17] Stern R., Kogan G., Jedrzejas M.J., Šoltés L. (2007). The many ways to cleave hyaluronan. Biotechnol. Adv..

[18] Andre B., Duterme C., Van Moer K., Mertens-Strijthagen J., Jadot M., Flamion B. (2011). Hyal2 is a glycosylphosphatidylinositol-anchored, lipid raft-associated hyaluronidase. Biochem. Biophys. Res. Commun..

[19] Csoka B.A., Scherer S.W., Stern R. (1999). Expression analysis of six paralogous human hyaluronidase genes clustered on chromosomes 3p21 and 7q31. Genomics.

[20] Stern R. (2004). Hyaluronan catabolism: A new metabolic pathway. Eur. J. Cell Biol..

[21] Yamamoto H., Tobisawa Y., Inubushi T., Irie F., Ohyama C., Yamaguchi Y. (2017). A mammalian homolog of the zebrafish transmembrane protein 2 (TMEM2) is the long-sought-after cell-surface hyaluronidase. J. Biol. Chem..

[22] Irie F., Tobisawa Y., Murao A., Yamamoto H., Ohyama C., Yamaguchi Y. (2021). The cell surface hyaluronidase TMEM2 regulates cell adhesion and migration via degradation of hyaluronan at focal adhesion sites. J. Biol. Chem..

[23] Yoshino Y., Goto M., Hara H., Inoue S. (2018). The role and regulation of TMEM2 (transmembrane protein 2) in HYBID (hyaluronan (HA)-binding protein involved in HA depolymerization/ KIAA1199/CEMIP)-mediated HA depolymerization in human skin fibroblasts. Biochem. Biophys. Res. Commun..

[24] Yoshida H., Okada Y. (2019). Role of HYBID (Hyaluronan Binding Protein Involved in Hyaluronan Depolymerization), Alias KIAA1199/CEMIP, in Hyaluronan Degradation in Normal and Photoaged Skin. Int. J. Mol. Sci..

[25] Yoshida H., Nagaoka A., Nakamura S., Sugiyama Y., Okada Y., Inoue S. (2013). Murine homologue of the human KIAA1199 is implicated in hyaluronan binding and depolymerization. FEBS Open Bio.

[26] Malaisse J., Evrard C., Feret D., Colombaro V., Dogné S., Haftek M., de Rouvroit C.L., Flamion B., Poumay Y. (2015). Hyaluronidase-1 Is Mainly Functional in the Upper Granular Layer, Close to the Epidermal Barrier. J. Investig. Dermatol..

[27] Chowdhury B., Hemming R., Faiyaz S., Triggs-Raine B. (2016). Hyaluronidase 2 (HYAL2) is expressed in endothelial cells, as well as some specialized epithelial cells, and is required for normal hyaluronan catabolism. Histochem. Cell Biol..

[28] Anderegg U., Simon J.C., Averbeck M. (2014). More than just a filler-the role of hyaluronan for skin homeostasis. Exp. Dermatol..

[29] Dokoshi T., Zhang L.-J., Li F., Nakatsuji T., Butcher A., Yoshida H., Shimoda M., Okada Y., Gallo R.L. (2020). Hyaluronan Degradation by Cemip Regulates Host Defense against Staphylococcus aureus Skin Infection. Cell Rep..

[30] Pepeliaev S., Hrudíková R., Jílková J., Pavlík J., Smirnou D., Černý Z., Franke L. (2017). Colorimetric enzyme-coupled assay for hyaluronic acid determination in complex samples. Eur. Polym. J..

[31] Meyer J.L., Stern R. (1994). Age-dependent changes of hyaluronan in human skin. J. Investig. Dermatol..

[32] Lin W., Shuster S., Maibach H.I., Stern R. (1997). Patterns of hyaluronan staining are modified by fixation techniques. J. Histochem. Cytochem..

[33] Cepa M. (2018). Segmentation of Total Cell Area in Brightfield Microscopy Images. Methods Protoc..

[34] Sinova R., Žádníková P., Šafránková B., Nešporová K. (2021). Anti-HA antibody does not detect hyaluronan. Glycobiology.

[35] Simek M., Hermannová M., Šmejkalová D., Foglová T., Souček K., Binó L., Velebný V. (2019). LC-MS/MS study of in vivo fate of hyaluronan polymeric micelles carrying doxorubicin. Carbohydr. Polym..

[36] Morgan T.W., Elson L.A. (1934). A colorimetric method for the determination of N-acetylglucosamine and N-acetylchrondrosamine. Biochem. J..

[37] Guntenhoner W.M., Pogrel M.A., Stern R. (1992). A substrate-gel assay for hyaluronidase activity. Matrix.

[38] Steiner B., Cruce D. (1992). A zymographic assay for detection of hyaluronidase activity on polyacrylamide gels and its application to enzymatic activity found in bacteria. Anal. Biochem..

[39] Richter W. (1974). Non-immunogenicity of purified hyaluronic acid preparations tested by passive cutaneous anaphylaxis. Int. Arch. Allergy Appl. Immunol..

[40] Ripellino J.A., Klinger M.M., Margolis R.U. (1985). The hyaluronic acid binding region as a specific probe for the localization of hyaluronic acid in tissue sections. Application to chick embryo and rat brain. J. Histochem. Cytochem..

[41] Tammi R., Ripellino J.A., Margolis R.U., Tammi M. (1988). Localization of epidermal hyaluronic acid using the hyaluronate binding region of cartilage proteoglycan as a specific probe. J. Investig. Dermatol..

[42] Atmuri V., Martin D.C., Hemming R., Gutsol A., Byers S., Sahebjam S., Thliveris J.A., Mort J.S., Carmona E., Anderson J.E. (2008). Hyaluronidase 3 (HYAL3) knockout mice do not display evidence of hyaluronan accumulation. Matrix Biol..

[43] Hatziri A., Vynios D.H., Panogeorgou T., Bouga H., E Triantaphyllidou I., Naxakis S.S., Stathas T., Aletras A.J., Kourousis C., Mastronikolis N.S. (2013). Presence of hyaluronidase isoforms in nasal polyps. Eur. Rev. Med. Pharmacol. Sci..

[44] Frost G.I., Csóka T.B., Wong T., Stern R. (1997). Purification, cloning, and expression of human plasma hyaluronidase. Biochem. Biophys. Res. Commun..

[45] Nawy S.S., Csóka A.B., Mio K., Stern R., Iozzo R.V. (2001). Hyaluronidase activity and hyaluronidase inhibitors. Assay using a microtiter-based system. Methods Mol. Biol..

[46] Boonen M., Puissant E., Gilis F., Flamion B., Jadot M. (2014). Mouse liver lysosomes contain enzymatically active processed forms of Hyal-1. Biochem. Biophys. Res. Commun..

[47] Puissant E., Gilis F., Dogné S., Flamion B., Jadot M., Boonen M. (2014). Subcellular trafficking and activity of Hyal-1 and its processed forms in murine macrophages. Traffic.

[48] Stern R., Jedrzejas M.J. (2006). Hyaluronidases: Their genomics, structures, and mechanisms of action. Chem. Rev..

[49] Harada H., Takahashi M. (2007). CD44-dependent intracellular and extracellular catabolism of hyaluronic acid by hyaluronidase-1 and -2. J. Biol. Chem..

[50] Zhang W., Yin G., Zhao H., Ling H., Xie Z., Xiao C., Chen Y., Lin Y., Jiang T., Jin S. (2021). Secreted KIAA1199 promotes the progression of rheumatoid arthritis by mediating hyaluronic acid degradation in an ANXA1-dependent manner. Cell Death Dis..

[51] Bourguignon V., Flamion B. (2016). Respective roles of hyaluronidases 1 and 2 in endogenous hyaluronan turnover. FASEB J..

[52] Papakonstantinou E., Roth M., Karakiulakis G. (2012). Hyaluronic acid: A key molecule in skin aging. Dermatoendocrinol.

[53] Csoka B.A., Frost G.I., Stern R. (2001). The six hyaluronidase-like genes in the human and mouse genomes. Matrix Biol..

[54] Ronny R., Mark M.E., Ceresa B. (2012). Hyaluronan Endocytosis: Mechanisms of Uptake and Biological Functions. Molecular Regulation of Endocytosis.

