# Supplementary material for: The Degradation of Hyaluronan in the Skin
Source: Biomolecules. 2022 Feb 3;12(2):251. doi: 10.3390/biom12020251 (PMC8961566; doi:10.3390/biom12020251)
Supplement: Supplementary file 1 [file biomolecules-12-00251-s001.zip › biomolecules-1535427-supplementary.pdf]

Supplementary Materials

The Degradation of Hyaluronan in the Skin

Petra Žádníková [1,2,†], Romana Šínová [1,3,†], Vojtěch Pavlík [1,4], Matěj Šimek [1], Barbora Šafránková [1], Martina Hermannová [1], Kristina Nešporová [1,*] and Vladimír Velebný [1]

[1] R&D Department, Contipro a.s., 561 02 Dolní Dobrouč, Czech Republic
[2] Faculty of Medicine in Hradec Králové, Charles University, 500 03 Hradec Králové, Czech Republic
[3] Institute of Experimental Biology, Faculty of Science, Masaryk University, 611 37 Brno, Czech Republic
[4] Third Faculty of Medicine, Charles University, 100 00 Prague, Czech Republic
* Correspondence: kristina.nesporova@contipro.com
† These authors contributed equally to this study.

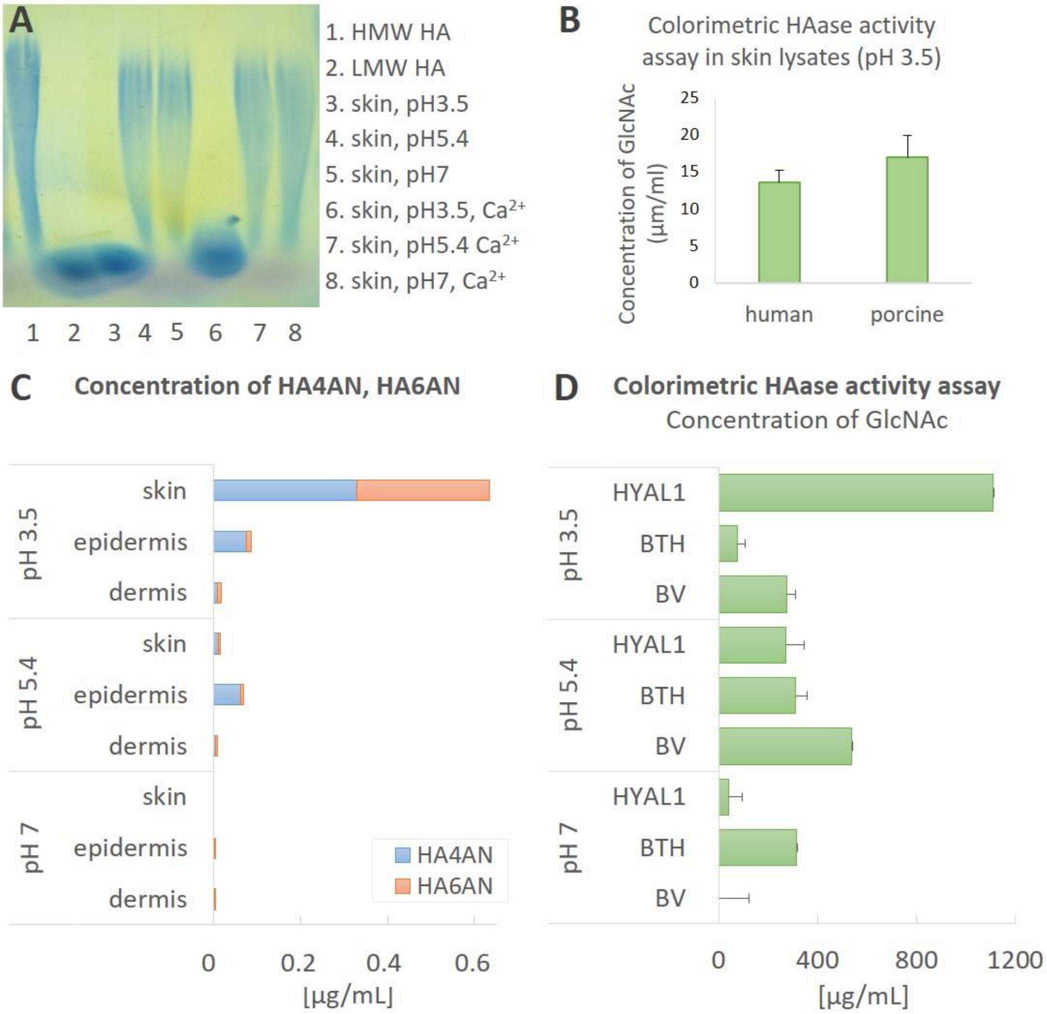

Figure S1. Hyaluronidase activity in the skin. Hyaluronidase activity in lysate from human skin (in the presence or absence of calcium ions and at the different range of pH) was detected by hyaluronan size analysis by agarose gel electrophoresis. Before analyses, lysates were mixed with exogenous HMW HA and incubated at 37 °C for 24 h (A). Comparison of hyaluronidase activity at low pH in human and porcine skin lysates (B). Concentration of HA4AN and HA6AN was detected (analytically by LC-MS/MS) in incubations mixtures (consist of lysates and HMW HA) and incubated at 37 °C for 24 h (C).

Hyaluronidase activity of selected enzymes (HYAL1, BTH, BV) was analyzed by colorimetric hyaluronidase activity assay, prior to analyses enzymes were mixed with HMW HA and incubated at 37 °C for 24 h (D). Three independent experiments were performed; data presented here are their averages. Grey bars represent the SEM of n = 3. Abbreviations: low molecular hyaluronan (LMW HA), high molecular hyaluronan (HMW HA), N-acetylglucosamine (GlcNAc), tetrasaccharides (HA4AN), hexasaccharides (HA6AN), hyaluronidase 1 (HYAL1), hyaluronidase from bovine testes (BTH), bee venom (BV).
